# Supplementary material for: Synonymous Codon Pair Recoding of the HIV-1 env Gene Affects Virus Replication Capacity
Source: Cells. 2021 Jun 29;10(7):1636. doi: 10.3390/cells10071636 (PMC8304268; doi:10.3390/cells10071636)
Supplement: Supplementary file 1 [file cells-10-01636-s001.zip › cells-1255049-supplementary.pdf]

```
                       *        20         *        40         *        60         *        80         *       100         *       120         *       140         *       160         *       180
WT_env      : ATGAGAGTGAAGGAGAAATATCAGCACTTGTGGAGATGGGGGTGGAGATGGGGCACCATGCTCCTTGGGATGTTGATGATCTGTAGTGCTACAGAAAAATTGTGGGTCACAGTCTATTATGGGGTACCTGTGTGGAAGGAAGCAACCACCACTCTATTTTGTGCATCAGATGCTAAAGCA
Max         : .....................................................................................................A........T..A...........C.....T........G..C.........T..........T..T.....A......
MaxCpG      : .....................................................................................................A........T..A...........C.....T........G..C.........T..........T..T.....A......
Max-3'WT    : .....................................................................................................A........T..A...........C.....T........G..C.........T..........T..T.....A......
MaxCpG-3'WT : .....................................................................................................A........T..A...........C.....T........G..C.........T..........T..T.....A......
Neu         : ...............................................................................................G..GC.............G..C..C........A...........G..C..T..T.....T..C.....C..T..C..C.....T
NeuCpG      : ...............................................................................................G..GC.............G..C..C........A...........G..C..T..T.....T..C.....C..T.....C.....T
Neu-3'WT    : ...............................................................................................G..GC.............G..C..C........A...........G..C..T..T.....T..C.....C..T..C..C.....T
NeuCpG-3'WT : ...............................................................................................G..GC.............G..C..C........A...........G..C..T..T.....T..C.....C..T.....C.....T
Min         : ..................................................................................................G........T...........C........C........A..G........T..A..G........G..T..C..C.....C
MinCpG      : ..................................................................................................G........T.....G.................T.....A..G........T..A..G........T..T.....A..G...
MinCpG.2    : ..................................................................................................G........T.....G...........G..A..C.....A..G........T..A..G........G..T.....C.....C
Min-3'WT    : ..................................................................................................G........T...........C........C........A..G........T..A..G........G..T..C..C.....C
MinCpG-3'WT : ..................................................................................................G........T.....G.................T.....A..G........T..A..G........T..T.....A..G...

                       *       200         *       220         *       240         *       260         *       280         *       300         *       320         *       340         *       360
WT_env      : TATGATACAGAGGTACATAATGTTTGGGCCACACATGCCTGTGTACCCACAGACCCCAACCCACAAGAAGTAGTATTGGTAAATGTGACAGAAAATTTTAACATGTGGAAAAATGACATGGTAGAACAGATGCATGAGGATATAATCAGTTTATGGGATCAAAGCCTAAAGCCATGTGTA
Max         : ........T..A.................A........A.....C........T..A..T..T..............A........C..T..G..C..C.......................G..G...........A..C..C.....CC.C.....C..G.....C.....C.....G
MaxCpG      : ........T..A.................A........A.....C........T..A..T..T..............A........C..T..G..C..C.......................G..G...........A..C..C.....CC.C.....C..G.....C.....C.....G
Max-3'WT    : ........T..A.................A........A.....C........T..A..T..T..............A........C..T..G..C..C.......................G..G...........A..C..C.....CC.C.....C..G.....C.....C.....G
MaxCpG-3'WT : ........T..A.................A........A.....C........T..A..T..T..............A........C..T..G..C..C.......................G..G...........A..C..C.....CC.C.....C..G.....C.....C.....G
Neu         : .....C..T.....G..C.....G.....G..G..C..G..C..G.....T........T........G..G...C.A..T.....T..T..G..C.....T........G...........G.....A...........C..T..A...C.T..............T..A..T.....G
NeuCpG      : .....C..T.....G..C.....G....................G.....T........T........G..G...C.A..T.....T..T..G..C.....T........G...........G.....A...........C..T..A...C.T..............T..A..T.....G
Neu-3'WT    : .....C..T.....G..C.....G.....G..G..C..G..C..G.....T........T........G..G...C.A..T.....T..T..G..C.....T........G...........G.....A...........C..T..A...C.T..............T..A..T.....G
NeuCpG-3'WT : .....C..T.....G..C.....G....................G.....T........T........G..G...C.A..T.....T..T..G..C.....T........G...........G.....A...........C..T..A...C.T..............T..A..T.....G
Min         : .....C..T........C.....G.....A..G.....G..C..G..A..C.....A.....T..G.....G..TC....T..C........G.....C..T...........C........T..G..A.....C........C..ATCCC..........GTCA..G..A..T..C..G
MinCpG      : .....C..T........C.....G.....A..C.....A..C..G..A..C.....A.....T..G.....G..TC....T...........G.....C..T....................T..G..A..............C..ATCCC..........G..T..G..A........G
MinCpG.2    : .....C..T........C.....G.....A..C...........G..A..G..T..A.....T..G.....G..TC....T...........G.....C..T....................T..G..A..............C..ATCCC..........GTCA..G..A..T.....G
Min-3'WT    : .....C..T........C.....G.....A..G.....G..C..G..A..C.....A.....T..G.....G..TC....T..C........G.....C..T...........C........T..G..A.....C........C..ATCCC..........GTCA..G..A..T..C..G
MinCpG-3'WT : .....C..T........C.....G.....A..C.....A..C..G..A..C.....A.....T..G.....G..TC....T...........G.....C..T....................T..G..A..............C..ATCCC..........G..T..G..A........G

                       *       380         *       400         *       420         *       440         *       460         *       480         *       500         *       520         *       540
WT_env      : AAATTAACCCCACTCTGTGTTAGTTTAAAGTGCACTGATTTGAAGAATGATACTAATACCAATAGTAGTAGCGGGAGAATGATAATGGAGAAAGGAGAGATAAAAAACTGCTCTTTCAATATCAGCACAAGCATAAGAGGTAAGGTGCAGAAAGAATATGCATTTTTTTATAAACTTGAT
Max         : ..GC.G..G..G........GTCC..G..A..T........A..A.....C..C..C..A...TCATCATCA..A........C..............A.....G......AGC.....C...TC...GTC...TC....A..A..A..A...........T............T.A...
MaxCpG      : ..GC.G..T..C........GTCC..G..A..T........A..A.....C..C..C..A...TCATCATCA..A........C..............A.....G......AGC.....C...TC...GTC...TC....A..A..A..A...........T............T.A...
Max-3'WT    : ..GC.G..G..G........GTCC..G..A..T........A..A.....C..C..C..A...TCATCATCA..A........C..............A.....G......AGC.....C...TC...GTC...TC....A..A..A..A...........T............T.A...
MaxCpG-3'WT : ..GC.G..T..C........GTCC..G..A..T........A..A.....C..C..C..A...TCATCATCA..A........C..............A.....G......AGC.....C...TC...GTC...TC....A..A..A..A...........T............T.A...
Neu         : ..GC.G......T.A.....GTCCC.G.....T.....CC.T..A.....C..A..C......TCA...TCT..C..G.................G..A..C..G.....T.....T.....ATCT..TTCT........C...........G..G..................T.G..C
NeuCpG      : ..GC.G......T.A.....GTCCC.G.....T.....CC.T..A.....C..A..C......TCA...TCT..C..G.................G..A..C..G.....T.....T.....ATCT..TTCT........C...........G..G..................T.G..C
Neu-3'WT    : ..GC.G......T.A.....GTCCC.G.....T.....CC.T..A.....C..A..C......TCA...TCT..C..G.................G..A..C..G.....T.....T.....ATCT..TTCT........C...........G..G..................T.G..C
NeuCpG-3'WT : ..GC.G......T.A.....GTCCC.G.....T.....CC.T..A.....C..A..C......TCA...TCT..C..G.................G..A..C..G.....T.....T.....ATCT..TTCT........C...........G..G..................T.G..C
Min         : ...C.G..A..GT.G..C..GTCAC.C..A........C..A.....C..C........A..CTC.TCGTCT..C........C.....A..G...........G..T..T..C..T..C..ATCG..GTCA..T.....C..A.....A..G.....C..C........C.....G...
MinCpG      : ...C.G..A..CT.G.....G...C.G..A........C..A........C........A..CTC.TCATCT..C........C.....A..G...........G..T..T..C..T..C..ATC....TCA..T..G..G..A.....A.....G........C.....C.....G...
MinCpG.2    : ...C.G..A..CT.G.....GTCAC.C..A........C..A........C........A..CTC....TCT..C........C.....A..G...........G..T..T..C..T..C..A.....T..T..T.....C..A...........G.....C........C.....G...
Min-3'WT    : ...C.G..A..GT.G..C..GTCAC.C..A........C..A.....C..C........A..CTC.TCGTCT..C........C.....A..G...........G..T..T..C..T..C..ATCG..GTCA..T.....C..A.....A..G.....C..C........C.....G...
MinCpG-3'WT : ...C.G..A..CT.G.....G...C.G..A........C..A........C........A..CTC.TCATCT..C........C.....A..G...........G..T..T..C..T..C..ATC....TCA..T..G..G..A.....A.....G........C.....C.....G...

                       *       560         *       580         *       600         *       620         *       640         *       660         *       680         *       700         *       720
WT_env      : ATAATACCAATAGATAATGATACTACCAGCTATAAGTTGACAAGTTGTAACACCTCAGTCATTACACAGGCCTGTCCAAAGGTATCCTTTGAGCCAATTCCCATACATTATTGTGCCCCGGCTGGTTTTGCGATTCTAAAATGTAATAATAAGACGTTCAATGGAACAGGACCATGTACA
Max         : ..T..T..T...........C..C..TTCA.....A..A...TCA.....T..T.....A..A..T..A.....C..C..A.....T........C..C.....C..C..C.....T..A..A..A.....C..C..C..G...........A..A..T.....C..G..C..G..C..C
MaxCpG      : ..T..T..T...........C..C..TTCA.....A..A...TCA.....T..T.....A..A..T..A.....C..C..A.....T........C..C.....C..C..C.....T..A..A..A.....C..C..C..G...........A..A..T.....C.....C.....C..C
Max-3'WT    : ..T..T..T...........C..C..TTCA.....A..A...TCA.....T..T.....A..A..T..A.....C..C..A.....T........C..C.....C..C..C.....T..A..A..A.....C..C..C..G...........A..A..T.....C..G..C..G..C..C
MaxCpG-3'WT : ..T..T..T...........C..C..TTCA.....A..A...TCA.....T..T.....A..A..T..A.....C..C..A.....T........C..C.....C..C..C.....T..A..A..A.....C..C..C..G...........A..A..T.....C.....C.....C..C
Neu         : ..T..T........C.....C..A..ATCA.....A.....TTCG..C..............A.....A........T..A..C..T........T..A..T........C.....G..T..A..C.....C..AT.G..G...........A..T..T.....G..G..T..C.....T
NeuCpG      : ..T..T........C.....C..A..ATCA.....A.....TTCC..C..............A.....A........T..A..C..T........T..A..T........C.....G..T..A..C.....C..AT.G..G...........A..T..T.....G.....T..C.....T
Neu-3'WT    : ..T..T........C.....C..A..ATCA.....A.....TTCG..C..............A.....A........T..A..C..T........T..A..T........C.....G..T..A..C.....C..AT.G..G...........A..T..T.....G..G..T..C.....T
NeuCpG-3'WT : ..T..T........C.....C..A..ATCA.....A.....TTCC..C..............A.....A........T..A..C..T........T..A..T........C.....G..T..A..C.....C..AT.G..G...........A..T..T.....G.....T..C.....T
Min         : ..C.....T..C..C.....C.....ATCG..C..AC.....TCG.....T..A..C..G...........A.....T..A..G..A..C.................C........G..T..G..A..C........C.....C...........T..T..C..G..T..C........C
MinCpG      : ..T.....T..T..C.....C.....ATCA..C..AC.....TCA.....T..A..G..G...........A..C..T......AG.....................C........A..T..A..A...........G.....C...........T..T.....C..T..C........C
MinCpG.2    : ..C.....T.....C.....C.....A.......................T...........A..C..A........T..A..G..A....................C........A..A.....C.....C.....C.....C...........T..T.....C..T..C........C
Min-3'WT    : ..C.....T..C..C.....C.....ATCG..C..AC.....TCG.....T..A..C..G...........A.....T..A..G..A..C.................C........G..T..G..A..C........C.....C...........T..T..C..G..T..C........C
MinCpG-3'WT : ..T.....T..T..C.....C.....ATCA..C..AC.....TCA.....T..A..G..G...........A..C..T......AG.....................C........A..T..A..A...........G.....C...........T..T.....C..T..C........C
```

```
                         *        740         *        760         *        780         *        800         *        820         *        840         *        860         *        880         *        900
WT_env       : AATGTCAGCACAGTACAATGTACACATGGAATTAGGCCAGTAGTATCAACTCAACTGCTGTTAAATGGCAGTCTAGCAGAAGAAGAGGTAGTAATTAGATCTGTCAATTTCACGGACAATGCTAAAACCATAATAGTACAGCTGAACACATCTGTAGAAATTAATTGTACAAGACCCAAC
Max          : .....TTCT..T...........T.....C..CC.C..G..G..C..C........A..AC.......TTC.T.............A........A...AG...T.....T..T..T.....A.....A...........A..A..T..T.....G..G..C..C..C..GC.G......
MaxCpG       : .....TTCT..T...........T.....C..C.....C..G..C..C........A..AC.......TTC.T.............A........A...AG...T.....T..T..T.....A.....A...........A..A..T..T.....G..G..C..C..C..T..G......
Max-3'WT     : .....TTCT..T...........T.....C..CC.C..G..G..C..C........A..AC.......TTC.T.............A........A...AG...T.....T..T..T.....A.....A...........A..A..T..T.....G..G..C..C..C..GC.G......
MaxCpG-3'WT  : .....TTCT..T...........T.....C..C.....C..G..C..C........A..AC.......TTC.T.............A........A...AG...T.....T..T..T.....A.....A...........A..A..T..T.....G..G..C..C..C..T..G......
Neu          : .....GTC......G..G..C..T..C..G..C........G...........G..A..TC.C......TCA.....T...........G......C.T..G........T..T........C.....T.....T..G.....A.....T..C..G..............TC.T.....T
NeuCpG       : .....GTC......G..G..C..T..C..G..C........G...........G..A..TC.C......TCA.....T...........G......C.T...........T..T........C.....T.....T..G.....A.....T..C..G..............TC.T.....T
Neu-3'WT     : .....GTC......G..G..C..T..C..G..C........G...........G..A..TC.C......TCA.....T...........G......C.T..G........T..T........C.....T.....T..G.....A.....T..C..G..............TC.T.....T
NeuCpG-3'WT  : .....GTC......G..G..C..T..C..G..C........G...........G..A..TC.C......TCA.....T...........G......C.T...........T..T........C.....T.....T..G.....A.....T..C..G..............TC.T.....T
Min          : .....GTCA..C..G...........C..C..AC.A..G..T..G.....C.....CT.AC.G..C...TCA..C..C..G...........G..C..G..C..G.....T..C........G..G..T..T.....C..A..C..T.....G..T..G...........CC.T..G..T
MinCpG       : .....GTCA..T..G..............C..A..A..G..T..G.....C......T.AC.T......TCA..T..G..............G..C..G.....G.....T..A........C..G..T..T.....G..A.....T........T..G...........C........T
MinCpG.2     : .....G..T.....C..............C..A........G..TAGT..C.....CT.AC.G......TCA.....G.....G..A..G..T..C..G..A........T..T........C..............C..A..C..T...AG...G.....C........C........T
Min-3'WT     : .....GTCA..C..G...........C..C..AC.A..G..T..G.....C.....CT.AC.G..C...TCA..C..C..G...........G..C..G..C..G.....T..C........G..G..T..T.....C..A..C..T.....G..T..G...........CC.T..G..T
MinCpG-3'WT  : .....GTCA..T..G..............C..A..A..G..T..G.....C......T.AC.T......TCA..T..G..............G..C..G.....G.....T..A........C..G..T..T.....G..A.....T........T..G...........C........T
```

```
                         *        920         *        940         *        960         *        980         *       1000         *       1020         *       1040         *       1060         *       1080
WT_env       : AACAATACAAGAAAAAGAATCCGTATCCAGAGAGGACCAGGGAGAGCATTTGTTACAATAGGAAAAATAGGAAATATGAGACAAGCACATTGTAACATTAGTAGAGCAAAATGGAATAACACTTTAAAACAGATAGCTAGCAAATTAAGAGAACAATTTGGAAATAATAAAACAATAATC
Max          : .....C..TC..........AA.A..A..A.....T..T...C.G........C..C..C..G..G..C..G..C.....G..G..G..C.....T..ATCA.................T...........A..T..CTC.....................G..C..C...........A
MaxCpG       : .....C..T...........AA.A..A..A.....T..T.....G........C..C..T..G..G..C..G..C.....G..G..G..C.....T..ATCA.................T...........A..T..CTC.....................G..C..C...........A
Max-3'WT     : .....C..TC..........AA.A..A..A.....T..T...C.G........C..C..C..G..G..C..G..C.....G..G..G..C.....T..ATCA.................T...........A..T..CTC.....................G..C..C...........A
MaxCpG-3'WT  : .....C..T...........AA.A..A..A.....T..T.....G........C..C..T..G..G..C..G..C.....G..G..G..C.....T..ATCA.................T...........A..T..CTC.....................G..C..C...........A
Neu          : ..T.....TC.G..GC.G...A.A...........G........G..C.....G..C..T..C..G..T..C..C.....G..G..C........T..A...C.T..............T...C....G..A..T..C..T.....G.....G..G.....C..C........T.....T
NeuCpG       : ..T.....T..G..G..G...A.A...........G........G..C.....G..C..T..C..G..T..C..C.....G..G..C........T..A.....G..............T...C....G..A..T..C..T.....G.....G..G.....C..C........T.....T
Neu-3'WT     : ..T.....TC.G..GC.G...A.A...........G........G..C.....G..C..T..C..G..T..C..C.....G..G..C........T..A...C.T..............T...C....G..A..T..C..T.....G.....G..G.....C..C........T.....T
NeuCpG-3'WT  : ..T.....T..G..G..G...A.A...........G........G..C.....G..C..T..C..G..T..C..C.....G..G..C........T..A.....G..............T...C....G..A..T..C..T.....G.....G..G.....C..C........T.....T
Min          : .........C.G...C.T..T..G..A...C.C..G.....C.....G..C..G..G.....C.....C..G......C.C.....G...........ATCCC.T..G..G.....C..T..AC.G........T..GTCT...C.GC.T.....G..C..G.....C.....G......
MinCpG       : ........T..G........TA.G..A.....G..G.....C.....C.....G........C........C..........................ATCC..G.....G.....C..T..AC.C........T..A..T...C.G........G.....C.....C.....C......
MinCpG.2     : ........T.....G......A.G..T........G.....C.....C.....G..T.....C.....T..C...........G..............A........C........C..T..AC.G...........CTCT...C.G.....G........C.....C.....C......
Min-3'WT     : .........C.G...C.T..T..G..A...C.C..G.....C.....G..C..G..G.....C.....C..G......C.C.....G...........ATCCC.T..G..G.....C..T..AC.G........T..GTCT...C.GC.T.....G..C..G.....C.....G......
MinCpG-3'WT  : ........T..G........TA.G..A.....G..G.....C.....C.....G........C........C..........................ATCC..G.....G.....C..T..AC.C........T..A..T...C.G........G.....C.....C.....C......
```

```
                         *       1100         *       1120         *       1140         *       1160         *       1180         *       1200         *       1220         *       1240         *       1260
WT_env       : TTTAAGCAATCCTCAGGAGGGGACCCAGAAATTGTAACGCACAGTTTTAATTGTGGAGGGGAATTTTTCTACTGTAATTCAACACAACTGTTTAATAGTACTTGGTTTAATAGTACTTGGAGTACTGAAGGGTCAAATAACACTGAAGGAAGTGACACAATCACCCTCCCATGCAGAATA
Max          : .....A...AGT..T.....T..............C..C.....C..C..C........A..G..C.......................T..C..C..C..G.....C..C..C..G..............AAGT.....T.................C.....G..G..C...C.....
MaxCpG       : .....A...AGT..T.....T..............C..C.....C..C..C........A..G..C.......................T..C..C..C..A.....C..C..C..G..............AAGT.....T.................C........G..C.........
Max-3'WT     : .....A...AGT..T.....T..............C..C.....C..C..C........A..G..C.......................T..C..C..C..G.....C..C..C..G..............AAGT.....T.................C.....G..G..C...C.....
MaxCpG-3'WT  : .....A...AGT..T.....T..............C..C.....C..C..C........A..G..C.......................T..C..C..C..A.....C..C..C..G..............AAGT.....T.................C........G..C.........
Neu          : .....A...AG...G..T.....T..T........G..C...TCC...........C..C..G..C..T..T.................A......TCA..A.........TCA..A...TCA.....G..CAGT.....T.....G..CTC......T..A..A..A..T..TC.....
NeuCpG       : .....A...AG...T..T.....T..T........G..C...TCC...........C..A..G..C..T..T.................A......TCA..A.........TCA..A...TCA.....G..CAGT.....T.....G..CTC......T..A..A..A..T..TC.....
Neu-3'WT     : .....A...AG...G..T.....T..T........G..C...TCC...........C..C..G..C..T..T.................A......TCA..A.........TCA..A...TCA.....G..CAGT.....T.....G..CTC......T..A..A..A..T..TC.....
NeuCpG-3'WT  : .....A...AG...T..T.....T..T........G..C...TCC...........C..A..G..C..T..T.................A......TCA..A.........TCA..A...TCA.....G..CAGT.....T.....G..CTC......T..A..A..A..T..TC.....
Min          : ........G.....T..C.....T..C..G..A..C..A...TC...C.....C..C..C..G........T.....C..T..C...........CTC...A.....C...TCC......TCG..G.....C.....C..T..C..G...TC......T..T..A..G.....TC.T..T
MinCpG       : ........G.....T..G.....T..T..G..A..C..A...TC...C........G..............T.....C..T..C...........CTC...A.....C...TCC......TCG..A..G..A.....C..T..G..G...TC......T..T..A..G.....T.....T
MinCpG.2     : ........G.....T..C.....T.....G..A..C..A...TC...C........C........C..T........C..T..C...........CTC...A.....C...TCC......TC...A..G..A.....C..T.....G...TC......T..T..A..G.....T..G..C
Min-3'WT     : ........G.....T..C.....T..C..G..A..C..A...TC...C.....C..C..C..G........T.....C..T..C...........CTC...A.....C...TCC......TCG..G.....C.....C..T..C..G...TC......T..T..A..G.....TC.T..T
MinCpG-3'WT  : ........G.....T..G.....T..T..G..A..C..A...TC...C........G..............T.....C..T..C...........CTC...A.....C...TCC......TCG..A..G..A.....C..T..G..G...TC......T..T..A..G.....T.....T
```

```
                         *       1280         *       1300         *       1320         *       1340         *       1360         *       1380         *       1400         *       1420         *       1440
WT_env       : AAACAAATTATAAACATGTGGCAGAAAGTAGGAAAAGCAATGTATGCCCCTCCCATCAGTGGACAAATTAGATGTTCATCAAATATTACAGGGCTGCTATTAACAAGAGATGGTGGTAATAGCAACAATGAGTCCGAGATCTTCAGACCTGGAGGAGGAGATATGAGGGACAATTGGAGA
Max          : ..G..G..C..C..............G..G..C..G..C...........G.....TTC.........A........T........A..T..TT.AT................A..A...TCA..T.....AAGT...........G..A........T..C...........C.....G
MaxCpG       : ..G..G..C..C..............G..G..C..G..C...........C.....TTC.........A........T........A..T..TT.AT................A..A...TCA..T.....AAGT...........G..A........T..C...........C.....G
Max-3'WT     : ..G..G..C..C..............G..G..C..G..C...........G.....TTC.........A........T........A..T..TT.AT................A..A...TCA..T.....AAGT...........G..A........T..C...........C.....G
MaxCpG-3'WT  : ..G..G..C..C..............G..G..C..G..C...........C.....TTC.........A........T........A..T..TT.AT................A..A...TCA..T.....AAGT...........G..A........T..C...........C.....G
Neu          : ..G..G..C.................G..T..C..G...........T..A......TCG..T......C.T.....T..C..C.....T..CT....TC.G..CC.T.....C..C..C.....T......AGT...........G..G...........C.....A.....C...C.T
NeuCpG       : ..G..G..C.................G..T..C..G...........T..A......TCG..T......C.T.....T..C..C.....T..CT....TC.G..C..G.....A..C..C.....T......AGT...........G..G...........C.....A.....C......
Neu-3'WT     : ..G..G..C.................G..T..C..G...........T..A......TCG..T......C.T.....T..C..C.....T..CT....TC.G..CC.T.....C..C..C.....T......AGT...........G..G...........C.....A.....C...C.T
NeuCpG-3'WT  : ..G..G..C.................G..T..C..G...........T..A......TCG..T......C.T.....T..C..C.....T..CT....TC.G..C..G.....A..C..C.....T......AGT...........G..G...........C.....A.....C......
Min          : ..G..G.....T..T........A..G.....C.....G........G.....G..A..C..C.....C..G..C.....C...........CT.AT.GC.G...C.C..C..C..G...TC...T..C.....A.....A.....G..A..C..C..G......C.C.........C..
MinCpG       : ..G..G.....T..T........A..G.....C.....T........A..C.....ATCA..C.....C..G..C.....C...........CT.AT.GC.G.....G.....G..G...TC...T........A.....A.....G..A..G..G..G.....................
MinCpG.2     : .....G.....T..T........A..G..G.....G..T...........A.....ATCA..C.....C..G..C.....C...........CT.AT.GC.G..T.....C..G..G...TC...T........A.....A.....G..A.....G..G........A............
Min-3'WT     : ..G..G.....T..T........A..G.....C.....G........G.....G..A..C..C.....C..G..C.....C...........CT.AT.GC.G...C.C..C..C..G...TC...T..C.....A.....A.....G..A..C..C..G......C.C.........C..
MinCpG-3'WT  : ..G..G.....T..T........A..G.....C.....T........A..C.....ATCA..C.....C..G..C.....C...........CT.AT.GC.G.....G.....G..G...TC...T........A.....A.....G..A..G..G..G.....................
```

```
                       *      1460         *      1480         *      1500         *      1520         *      1540         *      1560         *      1580         *      1600         *      1620
WT_env      : AGTGAATTATATAAATATAAAGTAGTAAAAATTGAACCATTAGGAGTAGCACCCACCAAGGCAAAGAGAAGAGTGGTGCAGAGAGAAAAAAGAGCAGTGGGAATAGGAGCTTTGTTCCTTGGGTTCTTGGGAGCAGCAGGAAGCACTATGGGCGCAGCCTCAATGACGCTGACGGTACAG
Max         : .....GC.C.......................A..G..GC.G..........................................................................................................................................
MaxCpG      : .....GC.C.......................A..G...C.G..........................................................................................................................................
Max-3'WT    : .....GC.C.......................A..G..GC.G..........................................................................................................................................
MaxCpG-3'WT : .....GC.C.......................A..G...C.G..........................................................................................................................................
Neu         : TCG.....G..............G..T........G................................................................................................................................................
NeuCpG      : TCG.....G..............G..T........G................................................................................................................................................
Neu-3'WT    : TCG.....G..............G..T........G................................................................................................................................................
NeuCpG-3'WT : TCG.....G..............G..T........G................................................................................................................................................
Min         : ..C..G..G..C.....C.....G..T.....C..G...C.C..........................................................................................................................................
MinCpG      : .....G..G..C.....C.....G..T.....A..G...C.T..........................................................................................................................................
MinCpG.2    : TC...G..G..C.....C.....G..T..G..A..G...C.G..........................................................................................................................................
Min-3'WT    : ..C..G..G..C.....C.....G..T.....C..G...C.C..........................................................................................................................................
MinCpG-3'WT : .....G..G..C.....C.....G..T.....A..G...C.T..........................................................................................................................................

                       *      1640         *      1660         *      1680         *      1700         *      1720         *      1740         *      1760         *      1780         *      1800
WT_env      : GCCAGACAATTATTGTCTGGTATAGTGCAGCAGCAGAACAATTTGCTGAGGGCTATTGAGGCGCAACAGCATCTGTTGCAACTCACAGTCTGGGGCATCAAGCAGCTCCAGGCAAGAATCCTGGCTGTGGAAAGATACCTAAAGGATCAACAGCTCCTGGGGATTTGGGGTTGCTCTGGA
Max         : ....................................................................................................................................................................................
MaxCpG      : ....................................................................................................................................................................................
Max-3'WT    : ....................................................................................................................................................................................
MaxCpG-3'WT : ....................................................................................................................................................................................
Neu         : ....................................................................................................................................................................................
NeuCpG      : ....................................................................................................................................................................................
Neu-3'WT    : ....................................................................................................................................................................................
NeuCpG-3'WT : ....................................................................................................................................................................................
Min         : ....................................................................................................................................................................................
MinCpG      : ....................................................................................................................................................................................
MinCpG.2    : ....................................................................................................................................................................................
Min-3'WT    : ....................................................................................................................................................................................
MinCpG-3'WT : ....................................................................................................................................................................................

                       *      1820         *      1840         *      1860         *      1880         *      1900         *      1920         *      1940         *      1960         *      1980
WT_env      : AAACTCATTTGCACCACTGCTGTGCCTTGGAATGCTAGTTGGAGTAATAAATCTCTGGAACAGATTTGGAATCACACGACCTGGATGGAGTGGGACAGAGAAATTAACAATTACACAAGCTTAATACACTCCTTAATTGAAGAATCGCAAAACCAGCAAGAAAAGAATGAACAAGAATTA
Max         : .....................................................AT.A.....A..A.....C.....C..G.......................A.....C.....GTCGC.C..T..T..A........G..GAGC..G.....A........A...............
MaxCpG      : .....................................................AT.A.....A..A.....C.....C..A.......................A.....C.....CTC.C.C..T..T..A........G..GAGC..G.....A........A...............
Max-3'WT    : ....................................................................................................................................................................................
MaxCpG-3'WT : ....................................................................................................................................................................................
Neu         : ..........................................TCA...........A..G.....A.....C.....A..A..............TC..........T.....T..TTCTC....T..T..AC.T..A..G..GAGT.....T..A.....G........G..G..GC..
NeuCpG      : ..........................................TCA...........A..G.....A.....C.....A..A..............T...........T.....T..TTCTC....T..T..AC.T..A..G..GAGT.....T..A.....G........G..G..GC..
Neu-3'WT    : ....................................................................................................................................................................................
NeuCpG-3'WT : ....................................................................................................................................................................................
Min         : ..........................................TCG.....G....................C..T..A..T........A.....T.....G..A...........TTCG..G..C..TAGTC.C..A..G.....C..............G..A..C..G.....G..G
MinCpG      : ..........................................TCC.....GAG..................C..T..A..T........A.....T.....G..A...........TTC...G..C..TAGTC.G..A..G.....C........A..G...........G..G...C.G
MinCpG.2    : ..........................................TC...C..G....................C..T..A..T........A.....T.....G..A........T...........C..TAGTC.C..A..G.....C........A..G...........G.....G..G
Min-3'WT    : ....................................................................................................................................................................................
MinCpG-3'WT : ....................................................................................................................................................................................

                       *      2000         *      2020         *      2040         *      2060         *      2080         *      2100         *      2120         *      2140         *      2160
WT_env      : TTGGAATTAGATAAATGGGCAAGTTTGTGGAATTGGTTTAACATAACAAATTGGCTGTGGTATATAAAATTATTCATAATGATAGTAGGAGGCTTGGTAGGTTTAAGAATAGTTTTTGCTGTACTTTCTATAGTGAATAGAGTTAGGCAGGGATATTCACCATTATCGTTTCAGACCCAC
Max         : ..A.................GTCGC.......C.....C.....C..C..C.....C.....C..C...C.T.....C.....T........A..A.....A...........A........G..C..C..T..A...C....AC.A..A..T..............A.....A......
MaxCpG      : ..A.................CTCAC.......C.....C.....C..C..C.....C.....C..C...C.T.....C.....T........A..A.....A...........A........G..C..C..T..A........A..A..A..T..............A.....A......
Max-3'WT    : ....................................................................................................................................................................................
MaxCpG-3'WT : ....................................................................................................................................................................................
Neu         : C.T.....G..C..G......TC.C.A.....C........T........C...........C..T.....G..T........T..G..G...C.T..G...C.CC.G..C..C.....G..C..AAG..........C....GC.......C............AGT............
NeuCpG      : C.T.....G..C..G......TC.C.A.....C........T........C...........C..T.....G..T........T..G..G...C.T..G...C.C..G..T..C.....A..C..AAG..........C....GC.......C............AGT............
Neu-3'WT    : ....................................................................................................................................................................................
NeuCpG-3'WT : ....................................................................................................................................................................................
Min         : ..A..G..G..C........CTCA..............C..T..T..T......T..........C...C.G..T........C..G.....G.....C..G..GC.T..T..G..C..G..T..G.....T...........GC.A........C..T..G..G...............
MinCpG      : C.T..G..G..C........C...C.............C..T..T..T......T..........C...C.G..T...........G..G..AC....T..G..GC.T..T..G........T..G.....T...........G..A.....G..C..T..C..G..C............
MinCpG.2    : ..A..G..G..C........CTCA..............C..T..T..T......T..........C...C.G..T...........G..G..TC.......G..G..G..T..G..C..G..T..G.....T...........G...........C..T..CC.TAGC............
Min-3'WT    : ....................................................................................................................................................................................
MinCpG-3'WT : ....................................................................................................................................................................................
```

```
                     *      2180         *      2200         *      2220         *      2240         *      2260         *      2280         *      2300         *      2320         *      2340
WT_env       : CTCCCAACCCCGAGGGGACCCGACAGGCCCGAAGGAATAGAAGAAGAAGGTGGAGAGAGAGACAGAGACAGATCCATTCGATTAGTGAACGGATCCTTAGCACTTATCTGGGACGATCTGCGGAGCCTGTGCCTCTTCAGCTACCACCGCTTGAGAGACTTACTCTTGATTGTAACGAGG
Max          : ....................................................................................................................................................................................
MaxCpG       : ....................................................................................................................................................................................
Max-3'WT     : ....................................................................................................................................................................................
MaxCpG-3'WT  : ....................................................................................................................................................................................
Neu          : ....................................................................................................................................................................................
NeuCpG       : ....................................................................................................................................................................................
Neu-3'WT     : ....................................................................................................................................................................................
NeuCpG-3'WT  : ....................................................................................................................................................................................
Min          : ....................................................................................................................................................................................
MinCpG       : ....................................................................................................................................................................................
MinCpG.2     : ....................................................................................................................................................................................
Min-3'WT     : ....................................................................................................................................................................................
MinCpG-3'WT  : ....................................................................................................................................................................................

                     *      2360         *      2380         *      2400         *      2420         *      2440         *      2460         *      2480         *      2500         *      2520
WT_env       : ATTGTGGAACTTCTGGGACGCAGGGGGTGGGAAGCCCTCAAATATTGGTGGAATCTCCTACAGTATTGGAGTCAGGAACTAAAGAATAGTGCTGTTAACTTGCTCAATGCCACAGCCATAGCAGTAGCTGAGGGGACAGATAGGGTTATAGAAGTATTACAAGCAGCTTATAGAGCTATT
Max          : ....................................................................................................................................................................................
MaxCpG       : ....................................................................................................................................................................................
Max-3'WT     : ....................................................................................................................................................................................
MaxCpG-3'WT  : ....................................................................................................................................................................................
Neu          : ....................................................................................................................................................................................
NeuCpG       : ....................................................................................................................................................................................
Neu-3'WT     : ....................................................................................................................................................................................
NeuCpG-3'WT  : ....................................................................................................................................................................................
Min          : ....................................................................................................................................................................................
MinCpG       : ....................................................................................................................................................................................
MinCpG.2     : ....................................................................................................................................................................................
Min-3'WT     : ....................................................................................................................................................................................
MinCpG-3'WT  : ....................................................................................................................................................................................

                     *      2540         *      2560
WT_env       : CGCCACATACCTAGAAGAATAAGACAGGGCTTGGAAAGGATTTTGCTA
Max          : ................................................
MaxCpG       : ................................................
Max-3'WT     : ................................................
MaxCpG-3'WT  : ................................................
Neu          : ................................................
NeuCpG       : ................................................
Neu-3'WT     : ................................................
NeuCpG-3'WT  : ................................................
Min          : ................................................
MinCpG       : ................................................
MinCpG.2     : ................................................
Min-3'WT     : ................................................
MinCpG-3'WT  : ................................................
```

Figure S1. Nucleotide sequence of CPB recoded HIV-1 *env* sequences designed and explored in this study. HIV-1 *env* regions that were no mutated in any variant are shown in blue. Substitutions for each variant are compared to the HIV-1 *env* HXB2 WT sequence.
